# Disruption of the NKG2A:HLA-E Immune Checkpoint Axis to Enhance NK Cell Activation against Cancer

**DOI:** 10.3390/vaccines10121993

**Published:** 2022-11-23

**Authors:** Jack G. Fisher, Amber D. P. Doyle, Lara V. Graham, Salim I. Khakoo, Matthew D. Blunt

**Affiliations:** School of Clinical and Experimental Sciences, University of Southampton, Southampton SO16 6YD, UK

**Keywords:** natural killer cell, NK cell, NKG2A, HLA-E, immunotherapy, checkpoint blockade

## Abstract

Ligation of the inhibitory receptor NKG2A by its ligand HLA-E negatively regulates the activation of natural killer (NK) cells, as well as subsets of CD8+ T cells and innate T cell populations. NKG2A has recently become a novel immune checkpoint target for the treatment of cancer and direct antibody mediated blockade of NKG2A function is currently under assessment in two phase 3 clinical trials. In addition to direct targeting, the NKG2A:HLA-E axis can also be disrupted indirectly via multiple different targeted cancer agents that were not previously recognised to possess immunomodulatory properties. Increased understanding of immune cell modulation by targeted cancer therapies will allow for the design of rational and more efficacious drug combination strategies to improve cancer patient outcomes. In this review, we summarise and discuss the various strategies currently in development which either directly or indirectly disrupt the NKG2A:HLA-E interaction to enhance NK cell activation against cancer.

## 1. Introduction

Disruption of inhibitory immune receptor signalling such as PD-1 and CTLA-4 on effector lymphocytes is now an established strategy for the treatment of cancer. More recently, targeting the inhibitory receptor NKG2A has been added to the list of viable immune checkpoint targets and holds promise for the treatment of solid and haematological malignancies. NKG2A, which binds the non-classical HLA molecule HLA-E, is expressed by subsets of natural killer (NK) cells, CD8+ T cells and innate T cells and negatively regulates their anti-tumour immune activity [1,2,3]. Disruption of NKG2A:HLA-E interactions are therefore of great interest to promote anti-tumour immunity and multiple different strategies are in development to directly target this immune checkpoint. In addition, NKG2A signalling can be disrupted indirectly via cancer therapies which were designed to target other pathways that are critical for cancer cell survival and growth. These agents therefore have a dual mechanism of action whereby NKG2A expressing immune effectors become activated against cancer, in addition to their cytostatic and cytotoxic properties. In this review we highlight the role of HLA-E and NKG2A in controlling immune cell function, focusing primarily on NK cells. We then describe the direct and indirect therapeutic modalities which disrupt NKG2A function and enhance anti-tumour immunity.

## 2. NKG2A and Control of NK Cell Activation

**Figure 1:**
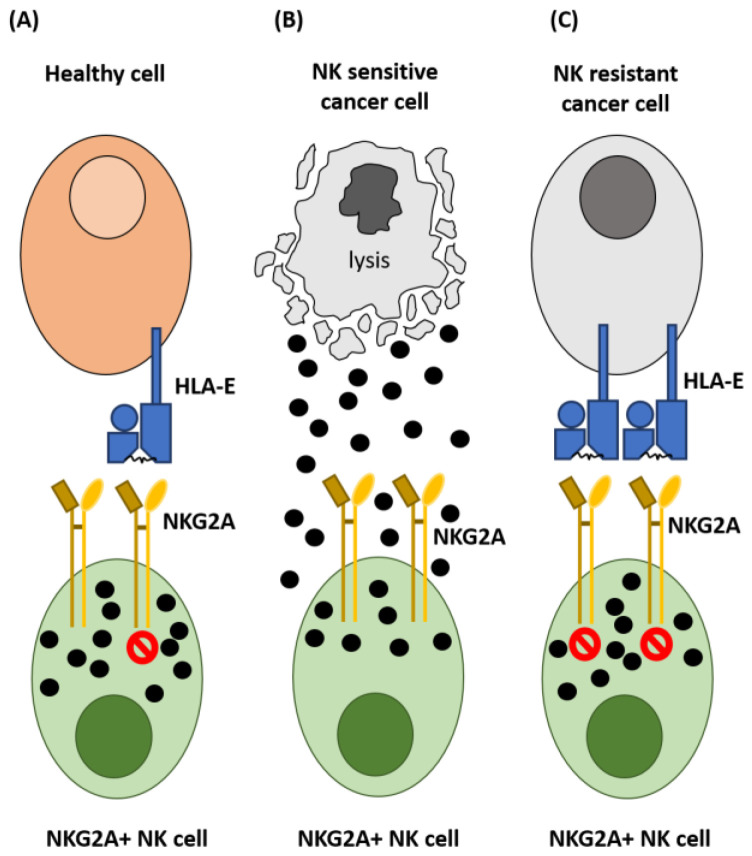
HLA-E:NKG2A regulation of NK cell cytotoxicity. (**A**) HLA-E presents HLA class I signal peptides to the inhibitory NK cell receptor NKG2A. HLA-E expression on nucleated, healthy cells negates NKG2A+ NK cell cytotoxicity toward healthy tissues. (**B**) Downregulation of HLA-E surface expression on stressed or abnormal cells makes them vulnerable to NKG2A+ NK cell mediated lysis. (**C**) Overexpression of HLA-E is frequently evident in cancers and can contribute to evasion of NK cell mediated detection and lysis.

NK cells are important for tumour control, with NK cell function, infiltration and frequency associated with outcome in numerous solid and haematological malignancies (reviewed in [4]). Their anti-cancer functions include secretion of cytotoxic granules, induction of death receptor signalling and production of inflammatory cytokines which drive adaptive anti-tumour immunity (reviewed in [4]). NK cells also contribute to monoclonal antibody therapy through their expression of CD16 (FcγRIIIA) and ability to mediate antibody-dependent cellular cytotoxicity (ADCC) [5,6]. NK cells are controlled by an array of activating and inhibitory receptors and their activation is dependent on a delicate balance between these signals. Activating receptors include NKp46, NKG2D, NKp44, NKp30, NKG2C and activating killer cell immunoglobulin-like receptors (KIR) (reviewed in [7,8,9]). The main inhibitory receptors expressed by NK cells are NKG2A and the inhibitory KIR [9]. Between 20–50% of circulating NK cells express NKG2A, with greater expression on CD56^bright^ NK cells compared to the CD56^dim^ NK cell subset [10]. HLA-E ligation with NKG2A on NK cells has been shown to disrupt actin formation at the immunological synapse negatively affecting their function (Figure 1) [11]. 

NKG2A is a member of the C-type lectin-like receptor superfamily, which includes the activating receptors NKG2D and NKG2C. Like NKG2C, NKG2A forms a heterodimer with CD94 and recognises the ligand HLA-E, however ligation of NKG2A transmits inhibitory signals via two immunoreceptor tyrosine-based inhibition motifs (ITIM) [12]. This recruits SHP-1/2 and leads to dephosphorylation of key activating signalling proteins such as VAV1, ZAP70 and src-family kinases resulting in NK cell inhibition [13,14]. NKG2A+ NK cells are sensitive to minute changes in HLA-E surface expression, indicating that partial disruption of NKG2A:HLA-E binding would be sufficient to unleash a potent anti-tumour immune response by NK cells [15]. Importantly, in addition to regulation of NK cell activation, NKG2A ligation is also required for NK cell education/licensing, a process by which NK cells become functionally competent during development [16] and thus long-term suppression of NKG2A signalling may compromise this process. Indeed, in murine models, knock-out of NKG2A results in defective NK cell cytotoxicity and IFNγ production [16]. NKG2A is also expressed on other effector lymphocytes including invariant natural killer T (iNKT) cells, activated CD8+ αβ T cells and γδ T-cells [17]. NKG2A ligation switches off T cell receptor signalling to dampen the immune response [12,18,19]. Conversely, NKG2A on Vδ2 T cells marks a sub-population of cells with enhanced anti-tumour effector functions [2]. 

In cancer, NK cell dysfunction has been described in multiple different cancer types [20,21,22] and stimulation of NK cell function and/or adoptive transfer of NK cells into cancer patients is of high clinical interest. Interestingly, a number of ex vivo expansion methods used for NK cell production potently increase NKG2A expression and NKG2A is also one of the first inhibitory receptors expressed after stem cell transfer [23,24,25]. In addition, high NKG2A expression predicts poor prognosis in a number of cancers including liver cancer [26] and head and neck cancer [27]. Patients with acute myeloid leukaemia (AML) present with higher NKG2A expression on peripheral NK cells compared to healthy controls and NKG2A expression correlates with failure to achieve remission [28,29]. In addition, compared to healthy controls, high expression of NKG2A is evident on defective NK cells isolated from patients with DLBCL [30]. Therapeutic strategies aimed at disrupting the NKG2A:HLA-E axis therefore have high potential for overcoming cancer mediated immune suppression in various solid and haematological cancer settings. Importantly, no health issues have been reported in mice deficient in CD94 and lacking NKG2A surface expression [31].

## 3. Expression of HLA-E in Cancer

The non-classical human leukocyte antigen-E (HLA-E) is constitutively expressed on nucleated cells at low levels and stabilisation of HLA-E at the surface membrane requires binding of HLA-E with peptides that are primarily derived from the leader sequences of classical HLA class I molecules and HLA-G [32,33]. Downregulation of HLA-A/B/C allows tumour cells to evade the immune system through reduced neoantigen presentation to cytotoxic T cells [34]. However, this can also lead to decreased surface HLA-E which enables recognition and destruction by NKG2A-expressing NK cells (Figure 1) [35,36]. Cancer cell immune evasion can also result from overexpression of HLA-E, even in the absence of HLA-A/B/C molecules (Figure 1) [37,38] and it has been noted that HLA-E may also bind peptides other than canonical leader sequences [39,40,41]. Importantly, the peptides bound to HLA-E influence NKG2A checkpoint blockade efficacy, with HLA-G peptide presentation by HLA-E on target cells associated with reduced NK mediated cytotoxicity compared with peptides derived from HLA-A/B/C [42]. Multiple solid and haematological malignancies have been observed to over-express HLA-E and associations between high HLA-E expression and worse clinical outcome have been reported in cancers including ovarian carcinoma, glioblastoma, gastric cancer and multiple myeloma [43,44,45,46].

High expression of HLA-E by tumour cells, tumour-associated macrophages and dendritic cells can limit the anti-tumour immune response by NKG2A+ CD8+ tumour-infiltrating lymphocytes [47]. CD8+ T cell infiltration and retained expression of classical HLA class I strongly associates with a better prognosis in non-small cell lung carcinoma (NSCLC), cervical and ovarian carcinomas, however high expression of HLA-E abrogates this predictive score, indicating that HLA-E influences resistance to T cell immune responses [48,49]. HLA-E and NK cell status serve as a sensitive prognostic factor in advanced gastric cancer [43], whilst HLA-E expression was identified as a major regulator of tumour cell susceptibility to NK cell lysis [50,51]. HLA-E expression can be increased on cancer cells by the presence of pro-inflammatory cytokines such as IFNγ that confer protection from NK cell lysis, demonstrating an immune escape mechanism in a pro-inflammatory environment [52,53]. Interestingly, HLA-E ligation of NKG2A can be used to improve cancer therapeutics in certain settings. For example, expression of HLA-E on allogenic primary NK cells prevents NK cell fratricide [54] whilst CD19-CAR T cells engineered with mutant B2M-HLA-E allowing constitutive expression of HLA-E protects against NK cell lysis [55].

## 4. Direct Inhibition of the NKG2A:HLA-E Interaction

**Figure 2:**
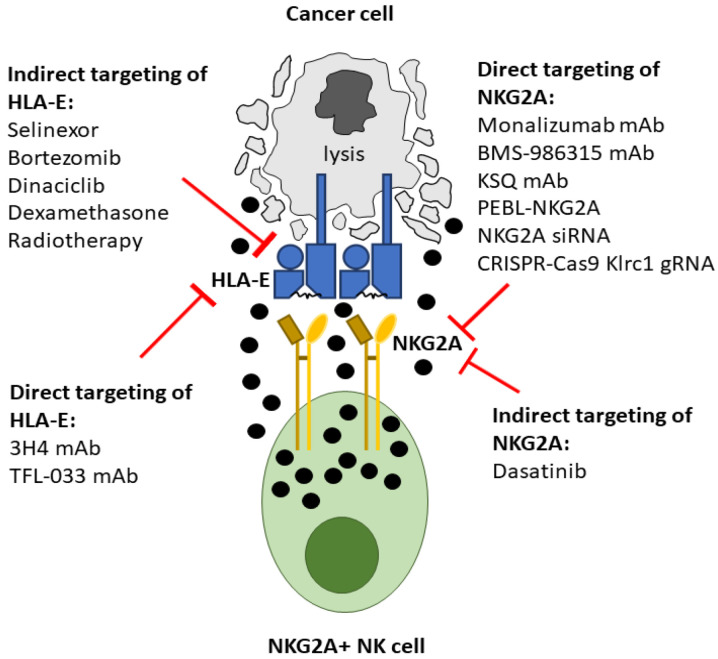
Therapeutic strategies to disrupt the inhibitory NKG2A:HLA-E axis and promote NK cell activation against malignant cells. Blockade of NKG2A:HLA-E interactions enhances cytotoxic granule release and cytokine production by NKG2A+ NK cells resulting in cancer cell lysis. Monoclonal antibodies (mAb) against HLA-E or NKG2A directly modulate NK cell effector functions by inhibiting the interaction of HLA-E and NKG2A. Modulation of NKG2A expression on NK cells also promotes NK cell anti-tumour functions. Expression of NKG2A at the plasma membrane can be downregulated by protein expression blockers (NKG2A-PEBL) which specifically constrain NKG2A in the Golgi. Genetic disruption to the NKG2A locus (*Klrg1*) can be achieved with CRISPR-Cas9 technology which inhibits its expression. Dasatinib, a tyrosine kinase inhibitor, also downregulates NKG2A expression leading to enhanced NK cell activity against tumours. Downregulation of surface HLA-E on tumour cells can be achieved with multiple different drugs that inhibit a variety of pathways. These include selinexor, a selective inhibitor of nuclear export; bortezomib, a proteasome inhibitor; dinaciclib, a pan-CDK inhibitor and dexamethasone, an activator of glucocorticoid receptors. Radiotherapy, which produces DNA damage can alter HLA-E expression on cancer cells.

The crucial role of NKG2A and HLA-E in dampening immune effector activation against cancer has made this inhibitory axis a key target for immunotherapeutic strategies. A number of different approaches are under development to directly target this checkpoint including antibodies, protein expression blockers and genetic engineering strategies (Table 1 and Figure 2).

## 5. Antibody Mediated Targeting of NKG2A

Immune checkpoint inhibitors (ICIs) have drastically improved cancer patient outcomes in multiple types of cancer [56]. The current approved ICIs target interactions between PD-L1:PD-1, CTLA-4:CD80/86 and LAG-3:MHC-II however not all patients respond and thus novel immune checkpoint inhibitors are under investigation [57,58,59]. Initial studies into the HLA-E:NKG2A interaction demonstrated that HLA-E transduction into the transformed B cell line 721.221 or peptide loading of HLA-E on RMA-S cells provided protection from NK cell-mediated lysis and this could be reversed using antibody blockade of NKG2A [1,60]. Furthermore, NKG2A blockade potentiated the activity of cytokine-activated, myeloma patient-derived NK cells against myeloma cell lines, demonstrating that anti-NKG2A mAbs can stimulate NK cells derived from cancer patients [61]. This is important because cytokine stimulation increases NKG2A expression on NK cells and acts as a brake on activation [61].

Monalizumab is a first-in-class anti-NKG2A IgG4 monoclonal antibody which significantly increases the degranulation and IFNγ production of NKG2A+ NK cells and activation of CD8+ T cells when co-cultured with K562 cells transduced with HLA-E and solid tumour cell lines [62]. Anti-NKG2A mAb as a monotherapy did not increase the survival of mice in a B-cell lymphoma pre-clinical model [62] and this lack of efficacy as a single agent was also reflected in clinical trials (NCT02459301) [63] (NCT03088059) [64]. However, monalizumab is well-tolerated by patients and is now being assessed as a combination therapy.

For instance, monalizumab and the anti-PDL-1 inhibitor durvalumab is currently undergoing phase II clinical testing for NSCLC with PD-1 immune checkpoint resistance (NCT03833440) and a phase III clinical trial for patients with stage III unresectable NSCLC (NCT05221840) [65]. The rationale for this combination derives from multiple studies showing that the NKG2A:HLA-E checkpoint associates with responsiveness to PD-1/PDL-1 blockade. For example, an in vivo CRISPR screen found that loss of Qa-1b (murine homolog of HLA-E) function was associated with increased efficacy of PD-1 immunotherapy [66] whilst a high frequency of PD-1+NKG2A+ CD8+ T lymphocytes were observed in head and neck squamous cell carcinoma (HNSCC), colorectal cancer (CRC) and lung cancer patients [62]. In addition, NKG2A associates with survival of bladder cancer patients treated with anti-PDL-1 antibodies and monalizumab increases the activation of CD8+ T cells derived from bladder tumours [67]. In a B cell lymphoma mouse model, the combination of NKG2A and PD-1 blockade rescued 75% of mice compared to 40% with durvalumab monotherapy [62].

In addition to enhancing the immune stimulating effect of PDx ICIs, monalizumab also potentiated the effect of direct targeting mAbs whereby NK cell-mediated ADCC with cetuximab (anti-EGFR) and obinutuzumab (anti-CD20) was enhanced by monalizumab [62]. Consequently, there is an ongoing phase III clinical trial assessing monalizumab in combination with cetuximab in patients with recurrent/metastatic HNSCC (NCT04590963). Importantly, NKG2A blockade has an acceptable safety profile in cancer patients [64] and in mice, anti-NKG2A administration led to partial depletion of HLA-E+ hematopoietic cells, but their numbers recovered 7 days post treatment [68].

NKG2A blockade also has potential for boosting the efficacy of cancer vaccines. For example, in a mouse model for HPV16-induced carcinoma, a therapeutic vaccine based on synthetic long peptides increased tumour cell expression of Qa-1b and increased tumour-infiltrating CD8+ T cell expression of NKG2A [27]. In accordance with this, the cancer vaccine in combination with NKG2A blockade doubled progression free survival in TC-1 tumour bearing mice [27]. This is of particular relevance as CD8+ NKG2A+ T cells constitute the majority of NKG2A+ cells in human lung cancer and are more prevalent than in the blood of patients and compared to healthy lung tissue [69]. NKG2A has recently been shown to be expressed as a late inhibitory checkpoint on proliferating CD8+ T cells repeatedly stimulated by antigen [3]. Thus, deploying anti-NKG2A mAbs may promote the activity (and memory formation) of highly specific anti-tumour cytotoxic T cells, making NKG2A an ideal candidate to target in combination with vaccines that induce prolonged TCR stimulation. Additionally, in contrast to PD-1, the absence of NKG2A on T regulatory cells avoids their activation, allowing further promotion of a pro-inflammatory environment [3,70].

In addition to monalizumab, other anti-NKG2A antibodies are currently in development including BMS-986315 [71] (NCT04349267) and a next-generation antibody (KSQ mAb) predicted to have higher binding affinity compared to monalizumab [72].

## 6. NKG2A Protein Expression Blockers (PEBL)

Ex vivo expanded NK cells express high levels of NKG2A [24,25,73] and this may result in reduced efficacy. Hence, strategies to modulate NKG2A expression on adoptively transferred NK cells in cancer are being actively investigated. One approach to achieve this is using an NKG2A protein expression blocker (PEBL) to circumvent elevated NKG2A expression on NK92 cells and healthy donor NK cells [25]. These PEBLs work by expressing a construct containing an anti-NKG2A domain derived from anti-NKG2A mAbs attached to an ER retention domain. This keeps the NKG2A protein in the Golgi and thence abrogates surface NKG2A expression. When transduced with NKG2A PEBLs, NK cells showed higher cytotoxicity against HLA-E expressing tumour cells and extended the survival of AML-bearing mice [25]. NKG2A-PEBL also enhanced NK cell ADCC against a breast cancer cell line opsonised with trastuzumab (anti-HER2 antibody) [25]. This approach shows promise for improving adoptive NK cell therapies and could be readily translated to adoptive T cell therapy strategies to prevent NKG2A expression on activated T cells [3].

## 7. NKG2A Small Interfering RNA

NKG2A knockout in expanded NK cells can also be achieved with small interfering RNAs (siRNA). These target NKG2A mRNA for degradation, thus inhibiting NKG2A protein translation and function, and can be beneficial through a number of different mechanisms. Primary NK cells transduced with NKG2A siRNA have been shown to be more cytotoxic towards an HLA-E positive B lymphoblastoid cell line [74] and in vivo adoptive transfer of NKG2A siRNA-transduced NKL cells enhanced clearance of HLA-E-positive 721.221 tumours [75]. Intriguingly, NKG2A siRNA also enhanced NK cell lysis of HLA-E negative K562 target cells, due to enhanced expression of the NK cell activating ligand NKp30 in NKG2A siRNA transduced NK cells [74]. Furthermore NKG2A knockdown with siRNA also enhanced the cytotoxicity of primary CD8+ T cells, demonstrating that NKG2A targeting siRNA technology could also be utilised in for T cell directed therapies [74].

Targeting NKG2A in vivo as opposed to ex vivo would circumvent the need for NK cell isolation, expansion and adoptive transfer in settings where patient NK cell function is not compromised. Recently, NKp46 antibody-coated lipid nanoparticles were used as vectors to deliver SHP-1 siRNA into NK cells [76]. These nanoparticles successfully downregulated SHP-1 and inhibited SHP-1-mediated signalling in NK cells [76]. This endowed NK cells with increased cytotoxic activity and when nanoparticles were used in a human B cell lymphoma mouse model, NK degranulation was enhanced with reduced tumour growth and improved survival [76]. Potentially, nanoparticles carrying NKG2A targeting siRNA could be used in tumour settings with high frequencies of functional NKG2A+ NK cells and could also be directed at NKG2A+ T cells. Although further investigations are needed to characterise the immune response using immune-targeting nanoparticles, the first siRNA nanoparticle therapy was approved in 2018 indicating that nanomedicine is now feasible [77,78].

## 8. CRISPR-Cas9 Gene Targeting of NKG2A

An alternative approach to abolish surface NKG2A expression on NK cells has been achieved using CRISPR-Cas9 technology. Guide RNAs specific for *Klrc1* (the NKG2A gene) were effective in reducing surface NKG2A expression in the NK-92 cell line by 50% [79]. However, this technology was less effective in IL-2 expanded primary NK cells, only reducing the NKG2A+ NK cell population by 17% [80]. However, in another study, targeting *Klrc1* in IL-15 expanded primary NK cells using CRISPR reduced the proportion of NKG2A+ cells by 50% [81]. The authors subsequently showed that following CRISPR-Cas9 gene editing, NK cells had higher cytotoxicity towards myeloma cell lines and primary patient myeloma cells. The cytotoxic capacity of these genetically engineered NK cells remained even after co-culture with IFNγ pre-treated myeloma cells with increased HLA-E expression. Partial abolition of NKG2A expression by CRISPR-Cas9 technology may be favourable as compared to complete NKG2A blockade as it may avoid deleterious effects on NK cell education [16].

## 9. Monoclonal Antibodies Targeting HLA-E

A potential issue with blocking NKG2A is the continuous recycling of NKG2A at the plasma membrane which will remove therapeutic mAbs from the tumour microenvironment [82]. In addition, long-term NKG2A blockade may interfere with the process of NK cell education and be detrimental to effector function [16]. An alternative approach is to target HLA-E directly. The non-polymorphic nature of HLA-E, and the high surface expression of HLA-E on tumour cells compared to healthy tissue makes it an attractive target. Development of antibodies which specifically recognise HLA-E epitopes and do not bind other HLA classes has been achieved and these have been shown to enhance NK cell and CD8+ T cell cytotoxicity against cancer targets [83,84].

Engagement of HLA-E with NKG2A is confined to a restricted subset of ‘VL9′ peptides with sequence VMAPRT(L/V)(V/L/I/F)L derived from the leader sequence of HLA class I molecules, in addition to certain viral derived peptides, providing protection from NK cell-mediated lysis [40,60,85]. When HLA-E is bound to peptides derived from other sources such as heat shock protein 60 (HSP60), this complex does not interact with NKG2A and enables an activating response by NK cells [86]. Hence, an antibody that specifically recognises HLA-VL9 complexes may allow efficient killing of HLA-E-VL9 expressing tumour cells whilst avoiding needless recognition of other HLA-E peptide complexes, particularly complexes that bind and activate NKG2C+ NK cells [87]. Li et al., (2022) isolated mouse and human anti-HLA-VL9 antibodies from naïve B cells which were shown to contact both HLA-E and the VL9 peptide. Interestingly, these antibodies were raised in non-immunised mice and isolated from human peripheral blood, implying the production of autoantibodies against HLA-E. The human antibody was of the IgM isotype and thus the lack of class-switching implies tolerance towards HLA-E-VL9. In vitro, these antibodies enhanced the cytotoxicity of the NKG2A-expressing NK-92 cell line against HLA-E-VL9 expressing target cells [84].

A potential advantage of anti-HLA-E mAbs over anti-NKG2A mAbs is their ability to exploit the ADCC capability of NK cells to further enhance the elimination of tumours. This resembles targeting of the PD-1/PD-L1 axis with avelumab, an anti-PD-L1 IgG1 mAb, whereby NK cell-mediated ADCC against PD-L1 expressing target cells can be achieved in tandem with blockade of PD-1 inhibitory signalling [88,89]. Utilising an anti-HLA-E mAb with CD16 binding properties may allow for activation of an ADCC response by NK cells against HLA-E expressing tumour cells, in addition to NKG2A signalling blockade.

**Table 1:** Direct targeting of NKG2A:HLA-E.

Therapeutic	Mechanism for Disruption of NKG2A:HLA-E Interactions	Clinical Stage	References
Monalizumab	Binds to NKG2A	Phase 3—HNSCC, NSCLC	[62]
BMS-986315	Binds to NKG2A	Pre-clinical	[71]
KSQ	Binds to NKG2A	Pre-clinical	[72]
3H4	Binds to HLA-E-VL9 complexes	Pre-clinical	[84]
TFL-033	Binds to HLA-E	Pre-clinical	[83]
NKG2A PEBL	Inhibits NKG2A expression by retaining NKG2A in the ER	Pre-clinical	[25]
NKG2A siRNA	Inhibits NKG2A expression by degrading NKG2A mRNA	Pre-clinical	[74,75]
CRISPR-Cas9 *Klrc1* gRNA	Targets *Klrc1* gene to downregulate NKG2A expression	Pre-clinical	[79,80,81]

## 10. Indirect Mechanisms for Inhibiting the NKG2A:HLA-E Interaction

HLA-E is expressed on the cell surface at much lower levels than classical HLA-A and -B molecules [90]. This implies that it can be lost readily from the cell surface, and a relatively small decrease in cell surface HLA-E can result in a large increase in NK cell activity [40]. Consistent with this, in viral infection the NKG2A:HLA-E receptor:ligand interaction is more readily targeted than KIR:HLA-C [91]. This precise balancing suggests that HLA-E may be susceptible to changes in cell metabolism/stress, and that these could be exploited for immunotherapeutic benefit. Thus, there are multiple studies demonstrating that cancer drugs which target a wide range of processes from nuclear export to proteasome degradation also disrupt this immune checkpoint (Table 2 and Figure 2).

## 11. Selective Inhibition of Nuclear Export

Exportin-1 (XPO1) transports key tumour suppressor proteins such as p53, retinoblastoma (Rb) and breast cancer 1 (BRCA1) from the nucleus to the cytoplasm, thereby inhibiting their functions [92]. XPO1 also regulates the export of ribosome constituents and oncogenic mRNAs to the cytoplasm and promotes translational activities [93]. Upregulation of XPO1 expression is evident in multiple solid and haematological malignancies [94] and selective inhibitors of nuclear export (SINEs) induce cancer cell apoptosis via restoration of tumour suppressor protein function and inhibition of oncoprotein translation [95]. The first-in-class XPO1 inhibitor selinexor has been approved for the treatment of relapsed and refractory multiple myeloma and diffuse large B cell lymphoma (DLBCL) and is currently under clinical evaluation in multiple solid and haematological malignancies.

It has recently been shown that XPO1 inhibition, in addition to being directly cytotoxic, downregulates HLA-E expression on malignant B cells and promotes NKG2A+ NK cell activation against lymphoma cells [96]. This effect is thought to be due to disruption of translation and a reduction in the availability of HLA-E stabilising peptides. In this model HLA-E forms highly unstable peptide complexes and is therefore sensitive to changes in the availability of de novo peptides [97,98]. This immunomodulatory property of XPO1 inhibitors may allow for combination strategies which both reduce nuclear export and increase susceptibility to NK cell attack. Importantly, XPO1 inhibition does not reduce NK cell numbers in vivo [99,100] and does not disrupt NK cell-mediated ADCC [101].

XPO1 inhibition potentiates ADCC against lymphoma cells in combination with anti-CD20 monoclonal antibodies rituximab and obinutuzumab in vitro [96] and combining selinexor with ADCC inducing antibodies may lead to increased efficacy in patients. Selinexor is currently in a phase 1 clinical trial for chronic lymphocytic leukaemia (CLL) in combination with the BTK inhibitor ibrutinib and the addition of NK cell adoptive transfer/CAR-NK cells and/or combination with an anti-CD20 antibody may further improve clinical responses (NCT02303392) [102,103]. Whether selinexor can also promote ADCC in combination with anti-CD38 antibodies such as daratumumab has not yet been investigated however represents an opportunity in multiple myeloma [104]. Interestingly, it has recently been shown that selinexor sensitivity in multiple myeloma patients is associated with expression of PVR, ULBP-1, -2 and -3 which are ligands for NK cell activating receptors [105]. In addition, another recent study identified that an IFNγ gene signature is associated with response to selinexor in multiple myeloma patients, pointing toward a possible NK cell involvement [106].

SINEs could also activate NK cells against other cancers. For example it was observed that selinexor downregulates surface HLA-E expression on monocytes 96, indicating that myeloid leukaemia’s may also be susceptible to NK activation in combination with selinexor. Adoptively transferred NK cells often have high levels of NKG2A+ NK cells and have been used to treat AML, and therefore SINEs may be an interesting combination for these studies in AML patients [107,108] (NCT00274846 and NCT01106950).

Whether SINEs also specifically activates NKG2A+ T cells against cancer cells has not been addressed although they can enhance the anti-tumour activity of CD19 directed CAR T cells against multiple lymphoma cell lines [109] and TRAIL-R2xCD3 bispecific antibodies against breast cancer cells [110]. In summary, XPO1 inhibition primes cancer cells for NK cell mediated cytotoxicity via HLA-E downregulation, and SINEs in combination with NK cell directed therapies has potential to promote anti-tumour responses in patients. Of note, CAR-NK cells show high expression of NKG2A and CAR-NK cells may therefore be ideal candidates for combination with selinexor [111].

## 12. Proteasome Inhibition

There are several proteasome inhibitors undergoing clinical testing with three currently approved for the treatment of multiple myeloma [112]. These compounds target different subunits of the proteasome complex, but all ultimately inhibit protein degradation by the proteasome. This inhibition leads to the accumulation of tumour suppressor proteins and cell cycle regulators which exert cytostatic and cytotoxic effects in cancer cells [113,114].

Bortezomib was the first proteasome inhibitor to be approved by the FDA and induces endoplasmic reticulum (ER) stress in multiple myeloma cells, leading to cell death [115,116]. Moreover, induction of ER stress by bortezomib renders myeloma cell lines sensitive to NK cells due to downregulation of surface HLA-E [98], potentially via decreased peptide loading [117]. In addition, proteasome inhibition has been reported to reduce HLA-E translocation to the cell surface. Thus bortezomib may also impact intracellular trafficking of HLA-E to the plasma membrane [118].

In addition to regulating HLA-E surface expression, bortezomib has also been shown to activate an anti-cancer NK cell response via upregulation of FAS and death receptor 5 (DR5) on leukaemia and colorectal cancer cell lines [119]. In a pre-clinical model of leukaemia, treatment with bortezomib plus NK cell adoptive transfer enhanced survival compared to either agent alone [119]. Utilisation of the immunomodulatory properties of proteasome inhibitors with combination strategies targeting NKG2A+ cells may overcome resistance mechanisms reported with bortezomib in patients [120,121].

## 13. Cyclin-Dependent Kinase Inhibition

Cyclin-dependent kinases (CDK) act in unison with cyclins to phosphorylate target proteins such as retinoblastoma (Rb) and drive cell cycle progression [122]. Activity of CDKs can become dysregulated during oncogenesis, and are the target of CDK-specific and pan-CDK inhibitors which induce cell cycle arrest and inhibit cancer cell proliferation [123]. In addition to their direct effects on the cell cycle, there are multiple studies showing that CDK inhibitors modulate the immune system. For example, the pan-CDK inhibitor, dinaciclib, which recently completed phase II clinical testing in AML (NCT03484520), reduces surface expression of HLA-E on AML cell lines and on primary AML cells, and promotes NK cell activation [124]. In addition, adoptive transfer of NK cells into dinaciclib-treated NSG mice reduced AML burden compared to controls, indicating the potential for combination therapy [124].

Three CDK4/6 inhibitors palbociclib, ribociclib and abemaciclib are FDA-approved for the treatment of advanced breast cancer [123] and whether these promote an NK cell immune response by modulating HLA-E expression has not been studied in detail. However, combination treatment of palbociclib and a MEK1/2 inhibitor in KRAS-mutant lung cancer has been shown to promote an NK cell immune response via upregulation of activating ligands ICAM-1, ULBP-2/5/6 and MICA [125]. Furthermore, an increased abundance of tumour-infiltrating NK cells was observed in a triple-negative breast cancer mouse model in the presence of CDK4/6 inhibitors and enhanced lymphocyte activation as measured by granzyme B expression was evident [126].

## 14. Dexamethasone

Dexamethasone is a corticosteroid used to suppress inflammation and is approved in combination with the anti-CD38 antibody daratumumab and bortezomib for the treatment of multiple myeloma [127]. Dexamethasone activates intrinsic apoptotic pathways in myeloma cells through multiple pathways including activation of the glucocorticoid response element, upregulation of pro-apoptotic gene expression and repression of NF-κB [128,129,130]. Additionally, dexamethasone initiates an NK cell immune response by downregulation of HLA-E surface expression on multiple myeloma cell lines [24]. Dexamethasone also downregulates surface expression of HLA class I proteins and increases surface expression of the NK cell activating ligands MIC-A/B, ULBP-1 and -2 and Fas [24]. In a murine model of multiple myeloma, adoptively transferred NK cells improved overall survival of mice in combination with dexamethasone [24]. Dexamethasone has undergone phase II clinical testing in multiple myeloma (NCT02626481) in combination with daratumumab [131] and the effect on NK cell ligands described above indicates that NK cell directed therapies in combination with dexamethasone may further promote tumour regression in multiple myeloma patients.

## 15. Tyrosine Kinase Inhibitors (TKI)

Dasatinib, along with other TKIs, is an ABL/SRC tyrosine kinase inhibitor used in the first-line treatment of chronic myeloid leukaemia (CML) and potently induces apoptosis of CML cells [132,133,134]. Incubation of NK cells with clinically relevant doses of dasatinib enhances NK cell mediated cytotoxicity and cytokine production against lymphoma and leukaemia cell lines [135] and also increases NK cell proliferation [136]. Dasatinib enhances NK cell cytotoxicity via downregulation of NKG2A, with no effect of dasatinib on other inhibitory NK cell receptors [137]. Interestingly the other two approved TKIs, imatinib and nilotinib, do not affect the expression of NKG2A [137], and in accordance with this, only dasatinib-treated CML patients showed enhanced NK cell cytotoxicity against target cells ex vivo [138]. Mechanistically, dasatinib induces downregulation of p38 MAPK, leading to reduced phosphorylation of GATA-3 and thus decreasing the activity of GATA-3 on the NKG2A promoter [137,139].

## 16. Radiotherapy

Radiotherapy induces DNA damage and apoptosis in cancer cells and can be used in combination with immunotherapy for the treatment of cancer [140]. Radiotherapy has also been shown to possess immunomodulatory activities [140] and can induce an NK cell immune response in the tumour microenvironment which is potentiated by DNA damage response inhibition [141]. Ionising radiation downregulated surface HLA-E expression on melanoma cells [142], however the effect of radiation on HLA-E expression appears to be dependent on tumour type and the model investigated. For example radiation increases HLA-E surface expression in glioblastoma [44] and also Qa-1b expression in murine models of melanoma and colorectal cancer [143]. Accordingly, NKG2A blockade improves survival in combination with radiotherapy in murine models of melanoma [143]. In contrast, radiotherapy has also been reported to reduce the sensitivity of cancer cells to NK cell mediated lysis [144] and it is therefore important to take this into account when considering strategies which combine radiotherapy and immunotherapy in patients.

**Table 2:** Indirect targeting of NKG2A:HLA-E.

Therapeutic	Direct Anti-Cancer Mechanism of Action	Mechanism for Disruption of NKG2A:HLA-E Interactions	Clinical Stage	References
Selinexor	XPO1 inhibitor	Downregulation of HLA-E	Approved MM, DLBCL	[96]
Bortezomib	Proteasome inhibitor	Downregulation of HLA-E	Approved MM, MCL	[98]
Dinaciclib	Pan-CDK inhibitor	Downregulation of HLA-E	Phase 2—multiple cancers	[124]
Dasatinib	Tyrosine kinase inhibitor	Downregulation of NKG2A	Approved—CML, ALL	[137]
Dexamethasone	Activation of the glucocorticoid response element, upregulation of pro-apoptotic gene expression and repression of NF-κB	Downregulation of HLA-E	Approved—multiple cancers	[24]
Radiotherapy	DNA damage	Downregulation of HLA-E/	Approved—multiple cancers	[142]
		Upregulation of HLA-E		[44]

## 17. Concluding Remarks

The NKG2A:HLA-E checkpoint is a highly promising and novel immune checkpoint target for the treatment of cancer. Antibody mediated blockade of NKG2A has an acceptable safety profile in patients and is currently under investigation in multiple clinical trials, including in two phase 3 trials in combination with cetuximab and durvalumab. In addition, various cytotoxic anti-cancer agents can unleash the activity of NKG2A+ NK cells which are highly sensitive to small changes in surface HLA-E expression on target cells. The immunomodulatory properties of these cytotoxic anti-cancer agents may allow for the design of more efficacious combination strategies. In this regard, NK cell therapies may be particularly applicable due to the increased NKG2A expression evident following various clinical-grade NK cell expansion techniques [24,25,73,145,146,147,148]. A precise understanding of the effect of these cytotoxic anti-cancer agents on NK cell viability/function directly, as well as on surface HLA-E kinetics and NK activating ligand expression on tumour cells will be required for optimal combination strategies.

## Data Availability

Not applicable.
